# Suicidal ideation and their relationship with job satisfaction and job strain among Jordanian hospitals’ healthcare professionals: a cross-sectional study

**DOI:** 10.3389/fpubh.2024.1393867

**Published:** 2024-05-17

**Authors:** Ann Mousa Alnajdawi, Rula Odeh Alsawalqa, Maissa N. Alrawashdeh

**Affiliations:** ^1^Department of Social Work, The University of Jordan, Aljubeiha, Jordan; ^2^Department of Sociology, The University of Jordan, Aljubeiha, Jordan

**Keywords:** suicidal ideations, job satisfaction, job strain, physician, nurse, Jordan

## Abstract

Suicidal ideation is a major health problem that occurs in response to complex interactions among psychological, sociocultural, and environmental factors. The phenomenon of suicidal ideation among healthcare professionals is still shrouded in mystery in Jordanian society, and there is still a scarcity of studies on its relationship with job strain and job satisfaction has been examined in the Arab world. Therefore, to fill this gap, this study aimed to reveal the prevalence of suicidal ideation among Jordanian hospitals health care professionals, and its relationship to job satisfaction and job strain. Additionally, how some sociodemographic factors were correlated with suicidal ideation levels, including age, sex, monthly income, occupation, years of experience, and sector type. Data were collected through a survey including sociodemographic characteristics, Suicidal ideation Scale (SIS), Job Satisfaction Survey (JSS), and Demand Control Support Questionnaire (DCSQ). The survey was distributed among 910 physicians and nurses of both sexes in public and private Jordanian hospitals. The survey was conducted using an anonymous online platform via Google Forms between October 2022 and December 2023. In light of the strain theory of suicide (STS), our results showed that physicians and nurses reported low levels of suicidal ideation and job satisfaction, with high levels of job strain. Suicidal ideation was positively correlated with job strain and negatively correlated with job satisfaction. Job satisfaction is negatively correlated with job strain. Job satisfaction was a significant mediator between job strain and suicidal ideation. Greater attention should be paid to the work environment in healthcare, particularly to enhance social support, increase job satisfaction levels, reduce job strain, and provide extensive training on effective prevention strategies for suicidal ideation and behaviour in the workplace. Additionally, policies and practices related to the health sector should be modified to create stable, healthy, and safe relationships and work environments.

## Introduction

Suicide is a complex and serious global public health problem among healthcare workers (1, 2). Recent studies have confirmed that healthcare workers, particularly physicians and nurses, are a high-risk suicide group (3, 4). Suicide is higher among physicians than in the general population, and the prevalence among female healthcare workers is higher than that among males (1, 2, 5–8). Among the factors that may increase the risk of suicide among physicians are work stressors, job problems (9), stressful long working hours, sleep deprivation, easy access to potentially fatal drugs, high levels of personal expectations, knowledge of lethal suicide methods (10), and aggressive and violent behaviour from patients (4). In addition, physicians are at high risk of burnout, which is associated with suicidal ideation (11).

Suicidal ideation (SI), Also called suicidal thoughts or ideas, is a broad term used to describe a range of contemplations, wishes, and preoccupations with death and suicide, SI definitions include thinking about planning suicide. SI is a heterogeneous phenomenon present in a waxing and waning manner and considered a better predictor of lifetime risk for suicide than imminent risk [(12): p. 1]. Experience suicidal ideations may occur to some people routinely, and to others only once in their lives. Ideations can range from a quick consideration to a detailed plan. Most people who experience suicidal ideations do not end their life, although some may make suicide attempts (13).

According to Dutheil et al. (4), the psychosocial work environment of physicians, including team conflicts, a high prevalence of shift work, lack of cohesive teamwork and social support, and constant dealing with unfortunate events and sad news, can explain the high risk of suicide among physicians. Moreover, weaknesses in personality traits and neuroticism, including sensitivity to other people’s opinions and criticism, compulsive attention to detail, exaggerated sense of duty, excessive sense of responsibility, and inability to seek help due to the culture of medical education, might contribute to physicians’ suicidal ideation (4, 11, 14).

The healthcare work environment is emotionally charged with the suffering and pain of patients and their families (11). Therefore, nurses are not immune to the pressures and problems faced by physicians. They suffer from emotional exhaustion (15), job strain, excessive workplace stress, lack of autonomy, work-related injuries, trauma, and work–family conflicts, which make them at a high risk of suicide (4, 16). Moreover, the work environments of nurses that include long, irregular hours, understaffing, workplace bullying, and verbal, physical, and sexual harassment may lead to suicidal ideation (16). Dobson et al. (17) found that suicidal ideation was prevalent among nurses.

Job strain is a stress situation that occurs as a result of physical and psychological pressures that the employee feels in the process of fulfilling what is expected by the workplace [(18): p. 777]. Job strain, stressful working demands, and long working hours of healthcare professionals raise the rate of occupational distress, anxiety, stress, and depression (4, 19, 20), and lead to low job satisfaction and thoughts of suicide (21). Job dissatisfaction increases the risk of suicidal ideation through increased anxiety and depression (14). Workers with a satisfactory level of job satisfaction are more likely to perform their work efficiently, increase their work productivity, and feel less hopeless and suicidal (22–24). Sarigül et al. (24) found that hopelessness and job satisfaction had parallel mediating effects on the relationship between work stress and suicidal cognition among healthcare workers. Psychological strain was positively correlated with suicidal ideation. Hopelessness and depression play serial mediating roles in the relationship between psychological strain and suicidal ideation (25). Among medical staff, Liu et al. (26) found that depressive symptoms were associated with longer working hours, less social support, value strain, aspiration strain, and coping strain. Furthermore, suicidal ideation was associated with longer working hours, coping with strain, and depression.

Psychache – psychological and emotional pain that reaches an intolerable intensity because there is no way out or it is an inescapable situation–is considered the primary factor causing suicide motivation. Furthermore, psychological strains cultivated in a social structure with negative life events, which need to be understood as intolerable pain with hopelessness, can be a necessary condition that creates a suicidal mentality, and a lack of social integration or low connectedness and high capability may all be sufficient conditions for suicide [(27): pp. 171, 173]. According to the strain theory of suicide (STS), conflicting and competing pressures in an individual’s life lead to suicide, which include (a) value strain from conflicting values, (b) aspiration strain from the discrepancy between aspiration and reality, (c) deprivation strain from relative deprivation such as poverty, and (d) coping strain from deficient coping skills in the face of a crisis (27). Based on the “ideation-to-action” framework, the Three-Step Theory (3ST) hypothesizes that suicidal ideation develops from the combination of pain and hopelessness. Connectedness is a key protective factor against escalating ideation among those experiencing pain and hopelessness. Moreover, (3ST) proposed that the progression from ideation to attempts is facilitated by dispositional, acquired, and practical contributors to the capacity to attempt suicide (28).

In Jordan, the phenomenon of physicians being abused and physical and verbal assaults at their workplace by patients is widespread, which may lead to murder, as happened in 2015, when a physician working in a government hospital in the capital of Amman was stabbed because of an argument started by a patient who refused to wait for his turn to receive treatment (29). Patients and their companions justify their violent behaviour towards physicians through medical errors, physician negligence, failure to provide adequate care, physician narcissism, lack of empathy, verbal miscommunication, and lack of sympathy in critical cases. In return, The Jordan Medical Association confirmed that healthcare problems were caused by a lack of medical staff and a disproportionate number of patients, obligating some medical staff to work more hours than permitted by law. Jordanian physicians indicated that the violence they faced, miscommunication, and inadequate interactions with their patients were caused by poor working environments, including complex hospital systems and shortages of medicine and medical equipment (30). In August 2022, a young physician committed suicide, after announcing her intention to commit suicide on social media sites, she jumped from the ninth floor of a private hospital in the capital Amman (29). The physician’s suicide preoccupied public opinion and raised concerns about the causes of her suicide, especially since those close to her confirmed that the pressures and poor living conditions she was experiencing, in addition to the pressures of long working hours for a small monthly salary, were among the main factors in her suicide (31).

The phenomenon of suicidal ideation among healthcare professionals is still shrouded in mystery in Jordanian society, especially among physicians and nurses, and there is still a scarcity of studies on its relationship with job strain and job satisfaction has been examined in the Arab world. Therefore, to fill this gap, this study aimed to reveal the prevalence of suicidal ideation among physicians and nurses in public and private Jordanian hospitals in Amman, and its relationship with the level of job satisfaction and job strain. We aim to understand the prevalence of suicidal ideation in light of important factors, including age, sex, monthly income, occupation, years of experience, and type of sector. Health care professionals, especially physician, are still among the groups with the least job satisfaction in Jordan. Jordanian physicians work long shifts, often for more than 40 h straight without adequate sleep or rest, for low pay or no pay at all, and they are exposed to traffic accidents due to exhaustion at work, and sometimes they die during work because of heart attacks that results of job stress, and many physician are exposed to bullying and insults by their managers as trainees (30, 32).

## Research questions

1. What is the prevalence of suicidal ideation among physicians and nurses in private and government Jordanian hospitals?
2. Is there a statistically significant association between suicidal ideation and the dimensions of job strain (psychological demands, decision latitude/control, and social support) among physicians and nurses?
3. Is there a significant association between suicidal ideation and Job Satisfaction among physicians and nurses?
4. Is there a statistically significant association between job satisfaction and dimensions of job strain (psychological demands, decision latitude/control, and social support)?
5. Are there differences between suicidal ideation among physicians and nurses due to age, sex, monthly income, occupation, years of experience, and sector type?
6. Does job satisfaction mediate the relationship between job stress and suicidal ideations**?**

## Materials and method

### Participants and data collection

Data were collected through a survey including sociodemographic characteristics, Suicidal ideation Scale (SIS), Job Satisfaction Survey (JSS), and Demand Control Support Questionnaire (DCSQ). The survey was distributed among 4,500 healthcare sector employees (physicians and nurses) of both sexes in public and private Jordanian hospitals via social media sites, such as WhatsApp, or email; access was through an anonymous online survey platform via Google Forms between October 2022 and December 2023. Social media sites and emails of participants have been verified through personal relationships with researchers and the hospitals’ official websites. Moreover, to ensure that participants met the inclusion criteria, demographic, professional and geographic characteristics were verified. This confirmed that every male or female physician and nurse were employed in public and private hospitals in Jordan, regardless of age, marital status, years of experience, and specialty.

Employees who consented to participate in the survey clicked “yes” on the first question in the survey, which was “Do you consent to participate in this study?” Once they selected this option, they were directed to complete the self-administered questionnaire, which typically took 10–20 min to complete. The questionnaire familiarized the participants with the objectives of this study. The online survey was conducted over approximately 12 months. Participants were informed that the data would be confidential and used only for scientific research purposes. The final sample size consisted of 910 participants. Table 1 presents the participants’ characteristics.

**Table 1:** Participants’ demographic characteristics (*N* = 910).

Demographics	*N*	%
Sex		
Male	545	59.9
Female	365	40.1
Age		
25–34	41	4.5
35–44	323	35.5
45–54	362	39.8
55 and over	184	20.2
Career		
Physician	430	47.3
Nurse	480	52.7
Monthly income		
Under 500 JD (1,057 $)	59	6.5
500–1,000 JD (1,059–1,410 $)	383	42.1
1,001–1,250 JD (1,411–1,763$)	274	30.1
1,251 JD and over (1,764$ and over)	194	21.3
Years of experience		
Less than five years	**39**	4.3
5-Less than 10 years	308	33.8
10-Less than 15 years	351	38.6
15 and over	212	23.3
Type of sector		
Public	584	64.2
Private	326	35.8

### Measures

#### Suicidal ideations scale

According to Luxton et al. (33), the 10-item Suicidal Ideations Scale [SIS; (34)] is a screening and assessment tool that provides critical information about the presence or absence of suicidal thinking, the intensity of those thoughts, and the presence or absence of prior suicide attempts. The SIS scored on a Likert five-point scale (1 = “Never,” 2 = “Infrequently,” 3 = “Sometimes,” 4 = “Frequently,” and 5 = “Always”). The total score ranged from 10 to 50. The SIS had a high level of internal consistency (Cronbach alpha = 0.86).

#### Job satisfaction survey

Developed by Spector (35) to measure job satisfaction among employees of human service organizations, it applies to all organizations. The JSS consists of 36 items with nine facets which are: Pay, Promotion, Supervision, Fringe Benefits, Contingent Rewards, Operating Procedures, Coworkers, Nature of Work, and Communication. The items are rated on a six-point Likert-type scale from “strongly disagree” to “strongly agree.” An Arabic translation of the survey was used from Paul Spector’s website (36).

#### The demand control support questionnaire

To measure job strain, the DCSQ, an established self-reported tool to measure a stressful work environment, was used. The 17-item DCSQ includes the three scales of psychological demands (five items), decision latitude/or control (six items), and social support at work (six items) Each item is rated on a 4-point Likert scale, “Strongly disagree,” “Agree,” “Disagree,” and “Strongly agree” (range, 1–4) (37–39).

### Data analysis

The collected data were analysed using the IBM (SPSS.V.25) Cronbach’s α value was used to estimate the internal consistency of the questionnaire for the present study. Descriptive statistics were used for demographic data. The Kolmogorov–Smirnov Test was used to determine whether the collected data were normally distributed. Pearson’s correlation coefficients were used to analyse the relationships between suicidal ideation, job satisfaction, and job strain. Moreover, one-way analysis of variance (ANOVA) was used to examine the differences in the arithmetic means of the respondents’ answers regarding suicidal ideation between healthcare professionals attributed to monthly income and number of years of experience. The Scheffé test was used for post-hoc comparisons of participants’ estimates of suicidal ideation. To examine the differences due to sex, occupation, and sector type, an independent-sample t-test was used. To reveal the mediating role of job satisfaction between job strain and suicidal ideation, path analysis was performed using the AMOS software.

## Results

Our measures showed excellent internal consistency between items a through Cronbach’s alpha coefficient (α); the suicidal ideations scale (α = 0.844), The job satisfaction scale (α = 0.900), and the job strain scale (α = 0.931). The Kolmogorov–Smirnov test results showed that the factors were normally distributed and all were greater than (0.05). For validity analysis, Pearson correlation analysis showed that the r value was significantly moderate (r = 0.32–0.84). The arithmetic means and standard deviations showed that the prevalence of suicidal ideation among healthcare professionals was low (M = 2.07, SD = 0.549), and their job satisfaction rate was low (M = 2.17, SD = 0.457). Participants reported high rates of job strain (M = 3.92, SD = 0.652), including psychological demands (M = 4.03), decision latitude/control (M = 3.96), and social support at work (M = 3.80) (see Table 2). Table 3 shows the results of Pearson’s correlation coefficient (r), which showed that suicidal ideation was positively correlated with job strain (r = 0.260) and negatively correlated with job satisfaction (r = 0.178). Job satisfaction was negatively correlated with job strain (r = 0.424) (Table 4).

**Table 2:** Mean and standard deviation of the prevalence suicidal ideation, job satisfaction and job strain among Jordanian hospitals health care workers.

Factors	M	SD
Suicidal ideation	2.07	0.549
Job satisfaction	2.17	0.457
Job strain	3.92	0.652
Psychological demands	4.03	0.714
Decision latitude/or control	3.96	0.789
Social support at work	3.80	0.652

**Table 3:** Pearson correlation coefficient analysis (*R*) between suicidal ideations and job strain, job satisfaction.

Factors	*R*	Suicidal ideations
Job strain	(*R*)	0.260**
	*Sig*	0.00
Psychological demands	(*R*)	0.205**
	*Sig*	0.00
Decision latitude/or control	(*R*)	0.256**
	*Sig*	0.00
Social support at work	(*R*)	0.224**
	*Sig*	0.00
Job satisfaction	(*R*)	−0.178**
	*Sig*	0.00

** Correlation is significant at the (0.01) level.

**Table 4:** Pearson correlation coefficient analysis (*R*) between job stress and job satisfaction.

Dimensions of job stress	*R*	Job satisfaction
Psychological demands	*(R)*	−0.342**
	*Sig*	0.00
Decision latitude/or control	*(R)*	−0.427**
	*Sig*	0.00
Social support at work	*(R)*	−0.349**
	*Sig*	0.00
Total	*(R)*	−0.424**
	*Sig*	0.00

**Correlation is significant at the (0.01) level (2-tailed).

The results of the independent-samples T-Test (Table 5) showed that there were no statistically significant differences at the level (α ≤ 0.05) for the scores of suicidal ideations due to the factors of sex (T = 1.789) and type of sector (T = 0.105). Statistically significant differences appeared due to occupation and were in favour of nurses (T = 9.499).

**Table 5:** Independent samples *T*-test results for differences in suicidal ideation scores due to factors (sex, type of sector, occupation).

Suicidal ideations					
Factors	M	SD	(*T*) calculated	(*T*) postulated	*Sig*
Sex					
Male	2.04	0.555	1.789	1.96	0.074
Female	2.11	0.538			
Type of sector					
Public	2.07	0.544	0.105	1.96	0.917
Private	2.07	0.558			
Occupation					
Physician	1.89	0.487	9.499	1.96	0.00
Nurses	2.22	0.555			

As shown in Table 6, the results of the One-Way ANOVA test revealed that there were statistically significant differences at the level (α ≤ 0.05) in the averages of participants’ responses toward suicidal ideations due to the monthly income factor (*F* = 7.481), in favour of those whose income was under 500 JD (1,057 $) according to the results of the Scheffe test. In addition, there were no statistically significant differences due to the factors of age (*F* = 1.621) and years of experience (*F* = 1.266).

**Table 6:** Results of the one-way ANOVA test to detect the differences in the scores of suicidal ideations due to the factors (monthly income, age, years of experience).

Suicidal ideations						
Source	SS	df	MS	(F) calculated	(F) postulated	Sig
Monthly income						
Between-group	6.616	3	2.205	7.481	2.60	0.00*
Within group	267.074	906	0.295			
Total	273.690	909				
Age						
Between-group	1.461	3	0.487	1.621	2.60	0.183
Within group	272.229	906	0.300			
Total	273.690	909				
Years of experience						
Between-group	1.142	3	0.381	1.266	2.60	0.285
Within group	272.548	906	0.301			
Total	273.690	909				

**Figure 1:**
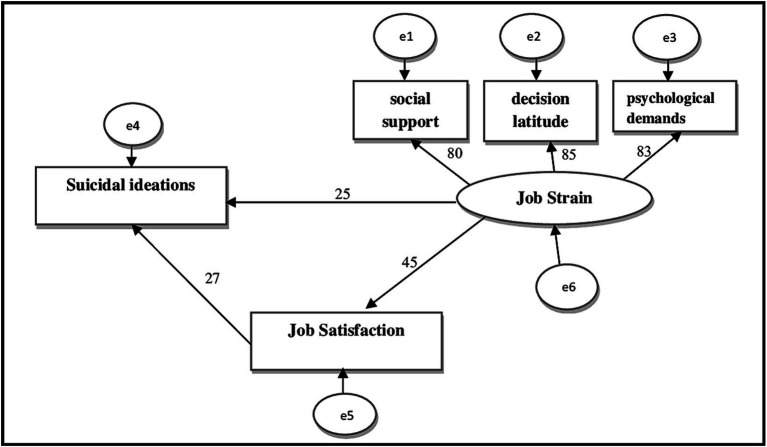
Path analysis (using AMOS) between measured factors in the present study.

As shown in Table 7 and Figure 1, path analysis using Amos software revealed a statistically significant relationship between job strain and suicidal ideation through job satisfaction as a mediating variable. This appeared through the chi-square test (Chi2), as the value reached (17.499) which is greater than its tabular and equal value (9.49), and through the test (CMIN/DF = 4.375) which was statistically significant (*p* = 0.002) and less than (0.05). The results also showed that the goodness-of-fit index (GFI = 0.992) was close to **1**; the closer it was to a true value, the better the quality of fit in the model (good-sufficient fit). The comparative fit index (CFI = 0.991) was also close to one, in addition to the root mean square error index (RMSEA = 0.061), which was close to zero and supported a good model fit (40). Job satisfaction (2.9%) was explained by the indirect relationship between job stress and suicidal ideation.

**Table 7:** Results of the path analysis test to verify the direct and indirect relationship between job strain and suicidal ideations in the presence of job satisfaction as an intermediate variable.

*Chi^2^*	Chi^2^ tabular	CMIN/DF	GFI	CFI	RMSEA	P	Path	Direct	Indirect	Total
17.499	9.49	4.375	0.992	0.991	0.061	0.002	Job strain → job satisfaction	−0.455	–	−0.455
							Job satisfaction→ suicidal ideations	−0.065	–	−0.065
							Job strain→ job satisfaction → suicidal ideations	0.248	0.029	0.277

## Discussion

Considering the increasing cases of violence and abuse against healthcare professionals, poor working conditions and environment, in conjunction with the poor economic situation, as well as increased anxiety and depression among working physicians and nurses, there is an increased possibility of heightened fear of the spread of suicidal ideation or suicide (41, 42). Olwan and Alsawalqa (43) confirmed that the number of Jordanians who committed suicide over the study period was 1,119, and most cases occurred in the capital city, Amman. Moreover, the most suicide factors were among unmarried, self-employed males in the 18–27 age group. Additionally, the main suicide risk factors were poverty, unemployment, high taxes, high cost of living, low wages, family disputes, failure, frustration, psychological problems/diseases, and emotional factors.

However, our results showed that the prevalence of suicidal ideation among physicians and nurses was low even though they reported high rates of job strain and low rates of job satisfaction. Conversely, suicidal ideation was positively correlated with job strain and negatively correlated with job satisfaction. While job satisfaction is negatively correlated with job strain.

In random sampling from the list of professionals (physician, nurse, other health professionals, technical, and administrative) in teaching hospitals, Oliveira et al. (44) found that there was a relationship between burnout and job satisfaction, and depression was a predictor of professional exhaustion. Psychosocial work factors play a major role in suicidal ideation, including quantitative and cognitive demands, low influence and possibilities for development, low meaning at work, low sense of community, role conflict, job insecurity, temporary employment, changes at work, and internal violence (45). Additionally, work-related stress is a significant predictor of suicidal cognition (24). Job stressors are determinants of common mental disorders and are associated with an elevated risk of suicidal ideation and behaviours (46), and there is a relationship between job strain, burnout, and suicidal ideation among workers in the healthcare sector (47). Kabir et al. (48) found a significant association between workplace bullying and burnout, and nurses’ suicidal ideation. Petrie et al. (49) used data collected as part of the 2013 Australian survey beyond the Blue National Mental Health Survey of Doctors and Medical Students. They found that work-related risk factors (perceived level of conflict between study, career, and family, personal responsibility, and bullying) are strongly associated with suicidal ideation. Fridner et al. (9) found that the role conflict was associated with suicidal ideation among physicians and that physicians with little control over working conditions had an increased risk of suicidal thoughts. Moreover, work support protected physicians when difficulties arose. Similarly, Akram et al. (50) found that life satisfaction is a negative predictor of suicidal ideation. Furthermore, the perceived life satisfaction holds a significant role in decreasing the levels of work–family conflict and suicidal ideation among physicians. While Howard and Krannitz (51) confirmed that the main factors that indirectly contribute to employees’ suicide attempts via depression and suicidal ideation are job autonomy, task variety, work–family conflict, family–work conflict, and job dissatisfaction. Our results showed that job satisfaction functioned as a significant mediator between job strain and suicidal ideation.

According to the strain theory of suicide, physicians still possess aspiration and coping skills in the face of crises, which prevents the formation of suicidal ideation. In a systematic review, Maresca et al. (52) found that the most common coping strategies among healthcare workers could be useful in preventing psychological suffering and burnout syndrome, particularly in stressful working conditions, including emotional and physical distancing from work, social and emotional support, physical self-care, and physical activity. Furthermore, physicians use several coping strategies, including acceptance, mindfulness, spirituality, socializing, provocative, and leisure activities, as well as emotional and problem-focused coping strategies, which are established habits of physicians [(53): p. 2]. Common coping strategies among nurses include social support and avoidance, spiritual entrust, exercising self-control, positive reassessment, emotion adjustment, help-seeking, communication coping, planful problem-solving, and self-improvement (54, 55). However, nurses use more problem-focused strategies than emotion-focused ones, such as positive reappraisal, problem-solving, planning, and acceptance [(56): p. 2]. The increased utilization of emotion-focused nurses was associated with increased job strain levels. These include escape avoidance, denial, venting, and symptom management coping strategies (56). Perceived control may be an important mediator of job stress (54).

In Jordan, Job satisfaction is considered a health indicator of work quality and emotional well-being or physiology. Working conditions, job security, availability of resources, work environment, and rewards are the most important determinants of job satisfaction among healthcare professionals (57). Work–life conflict is highly prevalent among Jordanian physicians. Work–life balance is important for supporting physicians’ well-being and performance. An increase in work-life balance is positively correlated with job satisfaction and life satisfaction among physicians, where long work hours negatively affect their lifestyles and time spent with their families (58). Psychological stress and depression are the primary causes of job dissatisfaction among physicians and nurses in Jordan. The major sources of psychological stress among physicians were the coordination of decisions within the team, impact of work on personal life, lack of time to perform the entire job, system of shifts, and lack of sleep. For Nurses, expectation of a call for help from patient, deal with poor working conditions, deal with cases of death, lack of sleep, and work long hours (59).

Özkan et al. (60) found a relationship between burnout dimensions (emotional burnout, depersonalization, and reduced personal accomplishment) and suicidal ideation among anaesthesiologists. Emotional exhaustion and reduced personal accomplishment diminish overall job satisfaction. Job dissatisfaction is a risk factor for mental health problems in workers (61). Lower job satisfaction is associated with less meaning in life, which in turn is associated with more symptoms of depression, anxiety, and anger (62). Workers with high levels of job dissatisfaction are more likely to develop depressive symptoms and suicidal ideation (61). Those with a satisfactory level of job satisfaction are much less likely to feel hopeless about their future and are more likely to perform efficiently in the workplace (24). Al Ma’aitah and Alsawalqa (15) confirmed that nurses in private hospitals in Amman, the capital of Jordan, suffered from emotional exhaustion, one of the dimensions of burnout, because they spent more time with patients. Moreover, their level of job satisfaction was low, especially for those working in paediatric departments and intensive care units (ICUs).

Regarding sociodemographic factors, our results found no statistically significant differences in the scores of suicidal ideation due to sex, type of work sector, age, and years of experience, while the monthly income variable was statistically significant (income under 500 JD (1,057 $)). Low income and economic inequalities could be potential predictors of suicidal ideation among healthcare professionals, owing to their negative effects on their quality of life and impeding career advancement (48). Kabir et al. (48) found that monthly income, geographical location of the workplace, department of work, and insufficient equipment to manage patients were associated with suicidal ideation among nurses. And nurses in the higher- and middle-income groups showed higher levels of suicidal ideation than those in the lower-income category. In contrast, Kim et al. (63) found that those in their 30s, 40s, and 60s who were divorced or widowed and those in their 50s and 60s who had never married or lived together were more likely to consider suicidal ideation. Except for those in their 20s and 80s, the rate of suicidal ideation tended to increase with lower household income and age.

In a systematic review, García-Iglesias et al. (64) confirmed that there is a set of factors that do not show a clear trend in studies of suicidal ideation and suicide attempts, such as age, sex, or type of healthcare professional. Some factors can be considered protective from suicidal tendencies and behaviours, particularly those related to the support system that the healthcare professionals have at the individual, family, and work level, such as dependent children, self-perceived social support, *per capita* income >3 minimum monthly wages, lower job-related stress, no family member infected, and years of service duration (more than 10 years).

Finally, Suicidal ideation is a major health problem that occurs in response to complex interactions among psychological, biological, environmental, and cultural factors (12). Healthcare professionals work in emotionally charged settings and have stringent job requirements. Consequently, they are more likely to experience stressful events and psychological disorders, which, if their intensity increases, with a lack of experience in coping strategies and an unhealthy work environment, especially in crises, could increase the likelihood of developing suicidal ideation (15, 65). Therefore, greater attention must be paid to the work environment in healthcare, particularly to enhance social support, increase job satisfaction levels, reduce job stress, and provide extensive training on effective prevention strategies for suicidal ideation and behaviour in the workplace. Additionally, policies and practices related to the health sector should be modified to create stable, healthy, and safe relationships and work environments.

This study is one of the first in the Arab world, especially in Jordan, to examine the prevalence of suicidal ideation among healthcare workers and its relationship to job satisfaction and job stress. The strengths of this study are its focus on healthcare workers, which is considered one of the most dynamic sectors and has multiple and overlapping job and emotional requirements. Furthermore, the study was conducted on an appropriate and diverse sample of physicians and nurses of varied sociodemographic characteristics. Moreover, it used measures with high validity and reliability, and was previously tested in an Arab and Islamic environment.

This study enriches the field of public health studies, occupational health and safety, and sociology of organization in Jordan and the Arab world and provides an understanding of the main problems of hospitals’ healthcare professionals. This allows decision makers to amend legislation and adopt new policies that contribute to improving the health care sector.

Nevertheless, the present study also had some limitations. First, it used convenience sampling due to the lack of a clear a sampling frame that allows choosing one of the random probability samples. Second, it would have been more suitable to add variables (age, sex, monthly income, occupation, years of experience, and sector type) in the path analysis. Finally, studying each sector type separately would have been better, given the different regulations in each of them, which may affect the results of the study, and reveal different factors and prevalence rates. However, the capabilities of researchers prevented this.

## Conclusion

Suicidal ideation is a complex and serious health problem among healthcare professionals and is considered a better predictor of lifetime risk for suicide than imminent risk. It has not been studied in the Jordanian health care sector; in Jordan and the Arab world, there is still a scarcity of studies on its relationship with job strain and job satisfaction. Further study is vital considering that the healthcare work environment is emotionally charged, healthcare professionals are still among the groups with the least job satisfaction in Jordan and work long shifts for low pay or no pay at all, and they are exposed to violence, bullying and insults by their managers or patients. This cross-sectional study aimed to reveal the relationship between suicidal ideation and their relationship with job satisfaction and job strain among physicians and nurses of both sexes in public and private Jordanian hospitals. The results showed that the prevalence of suicidal ideation among physicians and nurses was low even though they reported high rates of job strain and low rates of job satisfaction. In contrast, suicidal ideation was positively correlated with job strain and negatively correlated with job satisfaction; job satisfaction was negatively correlated with job strain. Additionally, there is no statistically significant differences in the scores of suicidal ideation due to sex, type of work sector, age, and years of experience, while the monthly income variable (under 500 JD (1,057 $)) was statistically significant. Our results highlight the importance of increasing job satisfaction, reducing job strain, raising wages commensurate with the cost of living and rising prices, and improving the infrastructure of the work environment in the healthcare sector. This would lead to improved psychological and mental well-being and the prevention of suicidal ideation among healthcare professionals in Jordan.

## Data availability statement

The original contributions presented in the study are included in the article/Supplementary material, further inquiries can be directed to the corresponding author.

## Ethics statement

The Institutional Review Board IRB at The University of Jordan granted ethical approval for this study in October 2022. Ref:19/2022/600.

## Author contributions

AA: Conceptualization, Data curation, Methodology, Project administration, Resources, Writing – original draft, Writing – review & editing. RA: Conceptualization, Data curation, Methodology, Project administration, Resources, Writing – original draft, Writing – review & editing. MA: Data curation, Methodology, Resources, Writing – review & editing.
